# Formulation and characterization of antibacterial orthodontic adhesive

**DOI:** 10.1590/2177-6709.24.4.073-079.oar

**Published:** 2019

**Authors:** Jefferson Twomley, Qingzhao Yu, Richard Ballard, Paul Armbruster, Xiaoming Xu

**Affiliations:** 1 Private practice (Asheville/NC, USA).; 2 Louisiana State University Health-New Orleans, School of Public Health, Biostatistics Program (New Orleans/LA, USA).; 3 Louisiana State University Health-New Orleans, School of Dentistry, Department of Orthodontics (New Orleans/LA, USA).; 4 Louisiana State University Health-New Orleans, School of Dentistry, Department of Comprehensive Dentistry and Biomaterials (New Orleans/LA, USA).

**Keywords:** Adhesive, Fluoride release, Antibacterial, Orthodontics

## Abstract

**Objective:**

The objective of this study was to formulate experimental orthodontic bracket adhesives and test their mechanical properties, fluoride release and antibacterial activity.

**Methods:**

Four experimental antibacterial orthodontic bracket adhesives were prepared with different compositions of synthesized antibacterial monomers replacing total 5% of dental monomers in the control Transbond XT (3M): 5%C11, 3.5%C11+1.5%C2, 5%C16, and 3.5%C16+1.5%C2. Transbond XT alone was used as control. These groups were used to bond premolar brackets to extracted premolars. Shear bond strength (SBS) was tested using an Instron machine. For antibacterial test, disk specimens (10mm diameter, 1mm thick, n=4) were fabricated and incubated with cultures of cariogenic Streptococcus mutans for 48h, and following gentle sonication, S. mutans biofilms in colony-forming-units (CFU) on the disks were enumerated by plating on agar medium. The data were analyzed using ANOVA and Tukey test (α=0.05).

**Results:**

All experimental groups had similar shear bond strength (no significant difference) to the control. All experimental groups showed significant inhibitory effect against S. mutans biofilm formation, when compared to the control, but there was no significant difference between experimental groups.

**Conclusion:**

Antibacterial orthodontic adhesive can be fabricated to have similar mechanical properties but better caries-inhibitory effect than current adhesive.

## RESUMO

**Objetivo:**

o objetivo do presente estudo foi formular, experimentalmente, adesivos para colagem de braquetes ortodônticos e testar as suas propriedades mecânicas, sua liberação de flúor e sua atividade antibacteriana.

**Métodos:**

quatro adesivos ortodônticos antibacterianos foram preparados experimentalmente usando monômeros sintéticos antibacterianos com diferentes composições, substituindo-se 5% do monômero do grupo controle (Transbond XT, 3M) por: 5%C11; 3,5%C11 + 1,5%C2; 5%C16; ou 3,5%C16 + 1,5%C2. O Transbond XT original foi utilizado como controle. Esses diferentes adesivos foram usados para colar braquetes de pré-molares em pré-molares extraídos. A resistência da colagem ao cisalhamento (RCC) foi testada utilizando-se uma máquina Instron. Para os testes antibacterianos, amostras em formato de disco (10 mm de diâmetro, 1 mm de espessura, n = 4) foram fabricadas e incubadas por 48 horas com culturas cariogênicas de Streptococcus mutans. Após leve sonicação, os discos contendo os biofilmes de *S. mutans* em unidades formadoras de colônia (UFC) foram colocados em placas com meio de cultura ágar e numerados. Os dados foram analisados utilizando-se ANOVA e teste de Tukey (α?#8197;= 0,05).

**Resultados:**

todos os grupos experimentais apresentaram RCC semelhante (sem diferença significativa) ao grupo controle. Todos os grupos experimentais apresentaram significativo efeito inibitório contra a formação do biofilme de S. mutans, em comparação ao grupo controle, porém sem diferença significativa entre os grupos experimentais.

**Conclusão:**

adesivos ortodônticos antibacterianos podem ser produzidos para alcançar propriedades mecânicas semelhantes às dos adesivos atuais, porém com melhores efeitos inibitórios de cáries.

## INTRODUCTION

One of the most frustrating challenges faced by orthodontists is the prevalence of enamel demineralization or white spot lesions (WSL). Even after a great occlusal and functional result has been achieved, the esthetics of orthodontic treatment can be marred by these opaque scars in up to 50% of patients.^1^ These lesions are a result of lowered pH due to the accumulation of biofilms of bacteria such as S. mutans.^2^ Even with a compliant patient, the mechanical removal of plaque and bacteria around orthodontic brackets is difficult, despite the patient’s best efforts. Because of this, fixed appliances can increase the accumulation of plaque and bacteria in orthodontic patients.^3^

One traditional method to combat demineralization and caries in general is the application of fluoride. The addition of fluoride during treatment with fixed appliances has shown to help reducing adhesion of bacteria, as well as their cariogenic activity.^4^ There are numerous ways to apply fluoride during treatment, such as varnishes and rinses. There have been many attempts to incorporate fluoride into adhesives, but these attempts can lead to a decrease in physical properties of the adhesive as well as a rapidly diminishing fluoride-releasing effect.^5^ ^,^ ^6^

Another way to combat the occurrence of WSL is to add other antibacterial properties to the orthodontic adhesives. Because of the erratic and often unreliable nature of patient compliance with hygiene, adding these properties provides another layer of protection for enamel that does not rely on the patient. Chlorhexidine has been added to bracket adhesive, but this can lead to a decrease in physical properties and has not shown significant reduction in demineralizations.^7^

The addition of nanoparticles has also helped to reduce the bacterial load around orthodontic brackets, particularly silver nanoparticles. There has been issues with dispersion and consistency with the mixing of the nanoparticles into the adhesive, but when mixed thoroughly, the result has shown good inhibition of bacteria without compromising physical properties.^8^ However, other studies have shown that incorporation of the nanoparticles decreases the bond strength of adhesives.^9^ *Galla chinensis* extract (GCE), a naturally-derived agent that has an inhibitory effect on bacteria, has also been added to resin-modified glass ionomer, with some success.^10^ Recent studies have also mixed an antibacterial QAS (quaternary ammonium salts) monomer, 2-methacryloxylethyl hexadecyl methyl ammonium bromide (MAE-HB), with orthodontic adhesive, producing promising results.^11^ ^,^ ^12^

The present research group has synthesized several antibacterial, fluoride-releasing monomers that can be added to different resins and adhesives. These antibacterial methacrylate or methacrylamide monomers contain long-chain quaternary ammonium fluoride. The dental composites and sealants containing these monomers have shown good antibacterial and fluoride releasing activity.^13,14,15^ The bactericidal mechanism of these antibacterial monomers with long-chain QAS is believed to be electrostatic interaction between the positive charge on QAS and negative charged bacteria membrane, which causes the disruption of the membrane and leak of cell contents, leading to the death of bacteria. Therefore, the general trend is that the longer the aliphatic chain in QAS, the stronger the interaction with bacteria and the higher the antibacterial efficacy. However, the longer chain of the antibacterial monomer can cause the reduction of mechanical properties of the polymer (cure composite and adhesive), because it increases the distance and reduces interaction between polymer chains. The monomers with long-chain QAS may also have poor miscibility with common dental monomers.

The purpose of this study was to formulate experimental orthodontic bracket adhesives using the synthesized antibacterial monomer with different chain lengths, and test their mechanical properties, fluoride release and antibacterial activity. The hypothesis is that adding antibacterial fluoride-releasing monomers to orthodontic bracket adhesives will increase fluoride release and reduce biofilm, while it will not adversely affect the mechanical properties of the adhesive.

## MATERIAL AND METHODS

### Material

**Figure 1:**
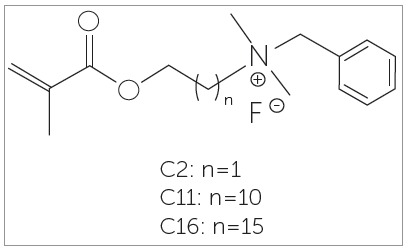
Structures of antibacterial monomers. Source: Reynolds^17^, 1975.

The three antibacterial monomers have been synthesized using a method similar to the previously reported.^13^ The structures of the monomers are shown in Figure 1. They contain the same polymerizable group (methacrylate) and alkyl-dimethyl-benzyl ammonium fluoride, a quaternary ammonium salt (QAS), but different lengths of aliphatic chain between the two functional groups.

To formulate the experimental adhesives, Transbond XT (3M Unitek, CA, USA) was mixed with three different monomers, which resulted in four experimental groups, replacing 5% of the Transbond XT: 5%C11, 3.5%C11+1.5%C2, 5%C16, 3.5%C16+1.5%C2. Transbond XT (TBXT) was used as the control group. 

### Fluoride release

Disk specimens (10mm diameter, 1mm thickness, n =5) were prepared for each group, light-cured for 40 seconds using an Optilux 501 dental curing light (Kerr, Orange, Calif., USA output >600 mW/cm^2^) and immersed in 3.0 mL deionized water at 37°C. The fluoride concentration of the solution was measured daily using an ion-selective electrode (model no. 96-09, Thermo Scientific Orion, Waltham Ma., USA) and 720 pH/ISE meter (Thermo Scientific Orion) for 28 days with daily replenishment of the solution.

### Antibacterial test

Disk specimens (10mm diameter, 1mm thick, n=4) of adhesives were fabricated (light-cured) and incubated with cultures of cariogenic Streptococcus mutans for 48 hours. The disk specimens were removed from the culture and placed in 3 ml of fresh BHI medium. After gentle sonication, the S. mutans suspensions of specimens were diluted by 10^3^ - 10^6^ times (into 10^-3^ - 10^-6^ of original concentrations) and drop-plated on BHI agar medium. After culture for 36 hours, S. mutans biofilms in colony-forming-units (CFU) on the agar medium were enumerated.

### Shear bond strength test

Fifty extracted mandibular premolars were randomly divided into five groups (n=10). Each was treated with the traditional pre-bonding method to enamel as follows: pumiced for 10 seconds, rinsed thoroughly, etched with 35% phosphoric acid gel for 30 seconds, rinsed thoroughly for 20 seconds, air dried, applied Transbond XT light-cure adhesive primer (3M Unitek, CA, USA) for 5 seconds, air dried for 5 seconds. Then, each group of adhesive was applied to the bracket base and the resin was pressed onto the enamel surface. The excess of adhesive was removed with an explorer and light-activated for 40 seconds (3M Unitek Ortholux™ Luminous Curing Light). All bonding procedures were performed according to the manufacturer’s instructions. The shear bond strength was tested on each group using an Instron 5566 Universal Testing Machine (Norwood, MA, USA) at a crosshead speed of 1mm/minute. The SBS was calculated into megapascals (MPa) using the formula MPa = F/A; where F is the maximum load, and A is bracket base area in mm^2^. The brackets used were mandibular premolar brackets, 0.022-in slot (Orthos, Ormco, Orange, CA, USA). Each tooth was embedded in self-cured acrylic resin with its long axis perpendicular to the horizontal plane.

### Statistical analysis

The data were analyzed using ANOVA and Tukey test (α=0.05).

## RESULTS

### Fluoride release

**Figure 2:**
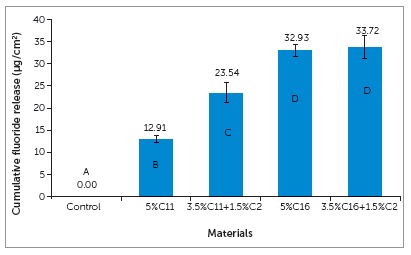
Cumulative fluoride release of experimental orthodontic bracket adhesives in 28 days (n=5, the groups with different letters have significant difference (p<0.05)).

**Figure 3:**
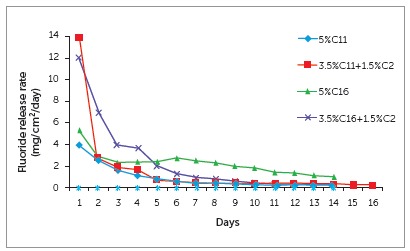
Fluoride release profile of experimental orthodontic bracket adhesives (n = 5).

All experimental groups showed fluoride release capabilities, while the control had no fluoride release, as shown in Figure 2. Both groups containing C16 had significantly more fluoride release than the groups containing C11. There was a significant difference between the C11 and C11+C2 groups, while the C16 and C16+C2 showed no significant difference. Each experimental group showed to continuously release fluoride over a 28-day period. Figure 3 shows a profile of fluoride release over a 14-day period.

### Antibacterial activities

**Figure 4:**
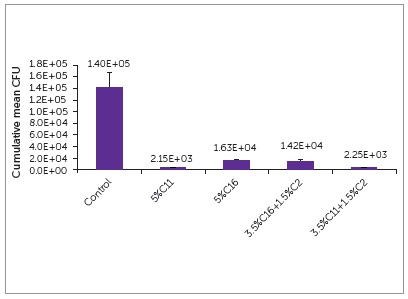
Antibacterial activities of experimental orthodontic bracket adhesives (n = 5). Lower CFU indicates higher biofilm inhibitory effect.

**Figure 5:**
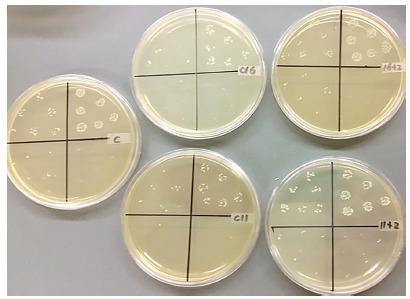
CFU of *S. mutans* biofilms from the adhesive samples when serial dilutions (10^-3^ - 10^-6^, counterclockwise) were spotted on BHI agar plates.

All experimental groups had significantly less colony forming units (CFU) than the control, as seen in Figures 4 and 5. Both C11 and C11+C2 had significantly less CFUs than both C16 and C16+C2. There was no significant change when C2 was added to either group.

### Shear bond strength

**Figure 6:**
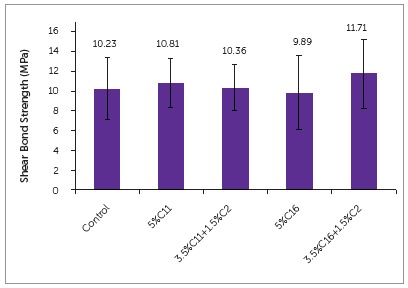
Shear bond strength of experimental orthodontic adhesives (n=10). There is no significant difference among different groups (p>0.05).

The mean shear bond strengths of all groups can be seen in Figure 6. The group C16 had the lowest bond strength (9.89MPa) and C16+C2 had the greatest bond strength (11.71MPa). However there was no statistical difference between any of the groups or the control. 

## DISCUSSION

The demineralization of enamel continues to be a concern for orthodontists with fixed appliances. The advent of direct bonding of orthodontic brackets simplified many aspects of treatment, but the problem of demineralization around the bracket continues to be a problem. As long as the problem persists, there will continue to be new ways to combat the problem.^16^

The addition of antibacterial monomers to orthodontic adhesive has shown to not affect the physical properties of the adhesive. The shear bond strength of the present experimental groups was similar to the control, at approximately 10 MPa. It has been recommended that orthodontic bracket adhesives have bond strength between 5.9 and 7.8 MPa to allow for sufficient bond to last the full length of treatment, while also allowing ease of removal upon debond.^17^ The bond strengths are equivalent to that of Transbond XT, which is the gold standard for orthodontic resin bonding.^18^ A recent meta-analysis of antibacterial orthodontic adhesive showed no decrease in shear bond strength, when compared to controls.^19^

As previously mentioned, the addition of silver nanoparticles can have dispersion issues when mixed with orthodontic adhesive, which leads to premature bond failures.^8,9^ While some studies have shown that the addition of these nanoparticles do not lower the SBS of the adhesive below what is clinically acceptable, there is a noticeable drop with the SBS. Degrazia et al.^9^ showed during their experiment that SBS remained above 15MPa for all experiment groups, but there was a 30-40% drop in bond strength when compared to the controls.

When compared to other experimental antibacterial orthodontic adhesives, the present experimental groups showed similar results. Fan et al^15^ formulated an experimental adhesive containing the monomer 2-methacryloxylethyl hexadecyl methyl ammonium bromide (MAE-HB), which produced SBS of approximately 10MPa, with no significant difference from the Transbond XT control. Altmann et al^20^ synthesized an experimental adhesive using 1,3,5-triacryloylhexahydro-1,3,5-triazine (TAT). In their experiment, shear bond strength actually increased when added to Transbond XT, compared to the control. This was believed to be due to copolymerization of the monomer with the TBXT. 

In addition to the antibacterial activity of QAS, the present monomer also has the benefit of releasing fluoride. Fluoride has traditionally been one of the most effective ways of preventing demineralization. It helps tip the balance away from demineralization towards remineralization. It has a cariostatic effect on S. mutans to help reduce bacterial load, as well as increasing enamel hardness and remineralization.^21^ When compared enamel surrounding traditional bracket adhesive, enamel surrounding a fluoride releasing adhesive was shown to have increased hardness, comparable to intact enamel, after exposure to cariogenic events.^22^ SEM studies also show more normal topography of enamel surrounding fluoride releasing resins, where enamel around traditional adhesives showed more erosion and roughening of the surface.^23^ The present experimental groups showed to have continuous release over 28 days, with an initial burst during the first 24 hours. The present C16 groups released more fluoride than the C11 groups and the control, with total fluoride release approximately 35 ppm over 28 days. The initial release was higher in the C11+C2 group (13.3 ppm) and lower in the C11 group (3.7 ppm). These ranges are comparable to glass ionomer cements tested by Chatzistavrou et al,^24^ and greater than fluoride releasing cements and resins tested by Regalla et al,^25^ which released 2-3 ppm in the first 24 hours and 9-15 ppm in the first 31 days. Both of the C16 groups continued to release between 0.5 and 1.5 µg/cm^2^/d. Rawls^26^ showed that releasing as little as 1.5 µg/cm^2^/d could inhibit demineralization. Dubroc et al^27^ showed that a fluoride releasing adhesive could reduce white spots up to 31% in mice with as little fluoride release as 0.5 to 1.0 µg/cm^2^/d. Although fluoride release is considered an important aspect in the adhesives of the present study, it should not lead to a reduction of the bond strength. Previous tests on commercially available fluoride releasing adhesives have shown a decrease in bond strength: Endo et al^28^ showed 30-40% reduction in the bond strength of a fluoride releasing resin, while Bishara et al^29^ showed a 40-55% reduction when comparing a glass ionomer fluoride releasing adhesive to Transbond XT. It has been shown that resin modified glass ionomer (RMGI) in general have lower SBS than resins, although they are still in an acceptable range.^30^ When choosing an adhesive for clinical use, it is important to keep in mind which ranges are clinically sufficient.^^

## CONCLUSIONS

» All experimental groups showed significantly greater ability to inhibit *S. mutans* colony formation than Transbond XT.
» All experimental groups showed significantly more fluoride release than the control, with the C16+C2 group showing the most.
» Adding different antibacterial monomers to Transbond XT resulted in shear bond strengths that had no statically significant difference to the Transbond XT alone.
» Therefore, such antibacterial monomers can be used to formulate antibacterial fluoride-releasing orthodontic adhesives that have good bond strength and additional benefit of reducing bacterial biofilm and white spot lesion.
