# ERRATUM

**DOI:** 10.1590/2177-6709.24.4.073-079.err

**Published:** 2019

The original article *“Formulation and characterization of antibacterial orthodontic adhesive”*, with DOI: 10.1590/2177-6709.24.4.073-079.oar, published in Dental Press J Orthod, vol. 24, no. 4, Jul./Aug. 2019, epub Sep 05, 2019, had in authorship the following authors and affiliations:

Jefferson Twomley^1^, Qingzhao Yu^2^, Richard Ballard^3^, Paul Armbruster^3^, Xiaoming Xu^4^

^1^Private practice (Asheville/NC, USA).

^2^Louisiana State University Health-New Orleans, School of Public Health, Biostatistics Program (New Orleans/LA, USA).

^3^Louisiana State University Health-New Orleans, School of Dentistry, Department of Orthodontics (New Orleans/LA, USA).

^4^Louisiana State University Health-New Orleans, School of Dentistry, Department of Comprehensive Dentistry and Biomaterials (New Orleans/LA, USA).

Now the article should have in authorship the following authors and affiliations: 

Jefferson Twomley^1^, Yapin Wang^4^, Zezhang Wen^2^, Qingzhao Yu^3^, 

Richard Ballard^4^, Paul Armbruster^4^, Xiaoming Xu^2^

^1^Private practice (Asheville/NC, USA).

^2^Louisiana State University Health-New Orleans, School of Dentistry, Department of Comprehensive Dentistry and Biomaterials (New Orleans/LA, USA).

^3^Louisiana State University Health-New Orleans, School of Public Health, Biostatistics Program (New Orleans/LA, USA).

^4^Louisiana State University Health-New Orleans, School of Dentistry, Department of Orthodontics (New Orleans/LA, USA).

